# Current News and Opinion

**Published:** 1886-01

**Authors:** 


### TWO OR THREE LITTLE THINGS.

You will often find the paper disks very handy for finishing proximal fillings in upper front teeth by hand. Take the disk between your thumb and finger, and you have a little file, sharp, flexible, and elastic.

---

The surgeons relieve hyperæsthesia by nerve-stretching. That is, they lay the nerve-trunk bare and forcibly stretch it. It is an old observation that wedging a tooth with proximal decay will frequently make it less sensitive, and I have supposed that this was because the wedge covered the opening of the cavity and excluded irritants. But two or three cases lately, in which this did not apply, have led me to think that, as the first effect of the wedge is to lift the tooth a little from its socket, we may have here a true case of relief from nerve-stretching.

---

Two drachms of menthol in an ounce of alcohol make an excellent application for a grumbling periodonteum, as Dr. Francis first taught me. But this solution is also the very best obtunder for softened and sensitive dentine that I have ever used. A drop in the cavity will take effect in three to five minutes, but sometimes a second drop is useful. It is effectual in about half the cases which need such help. J. SMITH DODGE, JR., M. D.

---

### DR. JAMES SHEPHERD.

The following resolutions of respect were passed at the twenty-first annual meeting of the Massachusetts Dental Society, Dec. 11th, 1885.

WHEREAS, The Massachusetts Dental Society hears with regret of the decease of our worthy and esteemed fellow member, Dr. James Shepherd, of Boston.

*Resolved*, That we hereby sympathize with his family and relatives in their sad bereavement, and that we tender to them the sympathy of this society, of which he was so long an honored member.

*Resolved*, That in his association with us he was ever wise, genial and kind, and won our private and professional esteem, and we shall miss his cheerful presence.

*Resolved*, That we were happy to know that in departing from us he had a firm faith in continued life, and a belief in the profound wisdom of Divine Providence, and we trust that in that Providence his friends and comrades will meet him again and renew with interest the friendships here formed.

*Resolved*, That a copy of these resolutions be forwarded to his family, and sent to the dental journals for publication.

Respectfully submitted,
J. T. CODMAN,
S. F. HAM,
S. B. JEWELL,
Committee.

Boston, Dec. 11, 1885.

---

### SURGEON BILLINGS.

At the time of the session of the last International Medical Congress, in Copenhagen, Dr. Billings, by order of the Secretary of War, attended it as the representative from the United States Army. The Treasury Department now refuses to allow his expenses, and he must pay them from his own pocket. This places us in a humiliating condition before other governments. The pay of a surgeon of the U. S. Army is not large, and it is an affront to the medical profession when a reasonable bill for such expenses is refused payment. It is not long since an admiral in the navy was forced to pay two hundred dollars from his own pocket for the entertainment of guests of the nation upon one of our national vessels.

---

Two Lion Cubs were lately born in the Philadelphia Zoological Gardens, but both died because of their inability to take nourishment, owing to their having cleft palate. "Aha!" says the Aqua Calcis dentist; "Another confirmation of my theory. They did not get a sufficiency of lime salts. The mother was fed on meat without the bones. There was a lack of material for bone making, and here is where the shortage was manifested."

If there had been a yet greater deficiency of lime, would the young lions have been without jaws at all, or would they possibly have had but three legs, or been lacking a tail? Really, the deficiency of lime should show in one bone as much as in another. But it so happens, in this case, that the chemical laboratory doctors had been heard in advance, and there was special pains taken during the gestation of the mother to see that she was supplied with bones in abundance, and phosphates in profusion. The case is but another confirmation of the fact that malformations of the mouth are very common in the young of wild animals of the feline species, when born in captivity.

---

The Medical and Surgical Reporter, without giving the name or place of residence of the physician, reports a terrible mistake recently made. A young woman suffered from an eye affection which necessitated enucleation. She was put under chloroform, and the operation performed, but it was found after she had recovered consciousness that the sound eye had been removed. The physician immediately fled the house, so thoroughly was he overcome by the mistake. A suit for malpractice will, of course, be forthcoming. Such a blunder is inexcusable under any conceivable circumstances.

---

Vaccination.—Persons entering the United States from Canada, if not already vaccinated, must submit to the process at the hands of the medical inspector upon the train. A lady, not long since, evaded the regulation by sewing a button inside the sleeve of her dress, in the usual place for vaccination. When the inspector demanded that she should bare her arm, she invited him to feel the scab of a previous vaccination, and presented the hidden button. The device succeeded, and the shrewd woman went on her way rejoicing.

---

A Crematory, built after the latest approved plans, is now under construction in Buffalo. It will be open for use as soon as finished, and then the ashes of Buffalonians can be decently inurned without sending the remains abroad. The crematory is built by an association of the physicians of the city.

Dr. Chisolm related two cases in which decided loquacity had been produced by the introduction of cocaine into a cavity in a decayed tooth. In speaking of cocaine tablets, he referred to a condition, amounting almost to a slough, that was produced on the buccal surface of his own cheek, from the application of one of these tablets to the gum and allowing it to remain till dissolved. From this, he hardly considered them the proper thing to use in nasal catarrh.—*Discussion in Baltimore Academy of Medicine.*

---

Mr. Oakley Coles, the well-known London dentist, writer, and society laborer, has abandoned dentistry for the Church. Mr. Coles is the author of a number of works of importance, and held many positions of trust and responsibility in the principal English dental societies. It is a matter of great regret that he should have decided to change his vocation, for he will be indeed fortunate if he finds himself in a position to do as much good to his fellow-men by preaching as by practice.

---

Dr. G. C. Daboll, of Buffalo, has retired from practice in this country, and proposes soon to sail for Paris, where he will make his permanent home. He has been engaged in practice in that city, and likes it, as well he may, for he met with unusual success there. It is not pleasant to part with so genial a companion and so good a friend, but—*Le Roi le veut.* It is but necessary that he should be himself to meet with success anywhere.

---

Dr. W. H. Morgan, of Nashville, Tenn., has received the unsolicited appointment of Commissioner of Indian Affairs, or Indian Agent. Had such men as Dr. Morgan always been selected for the management of our relations with the Indians, there would have been less of bloodshed, and our national reputation for fair and honorable dealing with these wards of the nation would not have been clouded as it is.

---

An Old Highlander, fond of his toddy, was ordered by his physician not to exceed one ounce of spirits daily. The old gentleman was rather dubious about the amount, and asked his son, a schoolboy, how much an ounce was. "Sixteen drams," was the reply. "What a guid doctor," exclaimed the Highlander; "run and tell Donald McTavish and Big John tae cam doon the nicht." —*Med. Review.*

---

M. Dujardin-Beaumetz has recently made a report to the French Academie de Medicine of a new hypnotic, or narcotic,—hypnone. Chemically, it is a phenyl-methyl-acetone, and it seems to possess remarkable soporific qualities. Given in doses of three or four drops, it produces a profound sleep. It has been given to a number of patients with no unpleasant effects.

---

Louis Elsberg.—The *New York Medical Journal*, for Dec. 5, contains an appreciative review of the life and professional work of Louis Elsberg, M. D., the well-known laryngologist.

WM. B. CARPENTER, C. B., M. D., LL. D., the well-known English physiolo gist and microscopist, is dead. Dr. Carpenter was, perhaps, more widely known than any other writer upon these subjects, and his writings have been adopted in almost every scientific school in which the English language is the mother tongue, as standard text books.

---

DR. KEITH, of Edinburgh, was recently brought from over the sea to give an opinion on the case of a lady, the cause of whose abdominal enlargement was obscure. He spent about half an hour with the case, and confirmed the diagnosis of the attending physician. For this service the Northwestern Lancet says he received $10,000.

---

DYSPEPSIA AND BAD TEETH are common companions. Their relations as cause and effect may be convertible, but that both are frequently due to the neglect of the teeth and their proper use will not be disputed by any one who is familiar with the physiological relations of these useful and much abused organs.—*The Sanitarian.*

---

MR. ARTHUR UNDERWOOD has been appointed editor of *The Journal of the British Dental Association.* Mr. Underwood is not unknown in this country, by his writings, and we are sure that he will grace the chair editorial. The association is to be congratulated upon securing the services of so competent a man.

---

J. H. SPAULDING, D. D. S., late of Minneapolis, has sailed for Europe, where he proposes to establish himself in practice. He has not as yet decided where he will locate. Dr. Spaulding has had some Old World experience, having practiced two years in Germany.

---

THE PROFESSOR OF ANATOMY in one of the medical colleges of this city recently asked a student what the sphenoid bone articulated with. He was answered: "With all the bones of the head except the coccyx, and in some rare instances with that."

---

THE NEW YORK MEDICAL JOURNAL relates a case in which the salicylate of cocaine was successfully used for the cure of persistent trigeminal neuralgia. In the course of six days, eight injections of six grains each into the cheek, produced permanent relief.

---

AN APOLOGY is due T. B. Welch & Co., whose corrected advertisement is in this number. The revision of their price list was sent us some time ago, but in the confusion which attended the removal of the editorial office it was lost. Note the changes.

---

TOO LATE FOR NOTICE in this number there was received a copy of the transactions of the Iowa State Dental Society's Twenty-third Annual Meeting. The volume presents a very handsome appearance.

